# Exploring the Syntheses for Phenolic Acid Esters of Cellulose: Pros and Cons of Methods

**DOI:** 10.1021/polymscitech.5c00028

**Published:** 2025-04-29

**Authors:** Thomas Elschner, Jakob Schönrich, Steffen Fischer

**Affiliations:** Institute of Plant and Wood Chemistry, 9169 Technische Universität Dresden, Pienner Str. 19, 01737 Tharandt, Germany

**Keywords:** cellulose, synthesis, phenolic acid ester, Mitsunobu, transesterification, *tert*-butyldimethylsilyl

## Abstract

Phenolic acid esters of cellulose show antioxidative and antimicrobial properties, which are very interesting for a variety of applications, including packaging and medicine. In contrast to simple mixing or encapsulation of phenolic compounds in polymeric materials, covalent linkage to the polymer backbone enables long-term activity and reduces toxicity because leaching of phenolic moieties is avoided. For the synthesis of such polymers, chemical modification of cellulose by polymer analogous reactions must be performed. Cellulose esters of ferulic-, 4-hydroxybenzoic-, vanillic-, protocatechuic-, and gallic acids are synthesized by two different approaches. On the one hand, transesterification of cellulose with corresponding methyl esters in DMSO/DBU/CO_2_ is appropriate to synthesize derivatives possessing only one phenolic hydroxyl group, e.g. hydroxybenzoates and vanillates. On the other, conversion with TBS-protected imidazolides is carried out, which allows the synthesis of cellulose esters possessing multiple phenolic hydroxyl groups such as protocatechuates and gallates.

## Graphical abstract

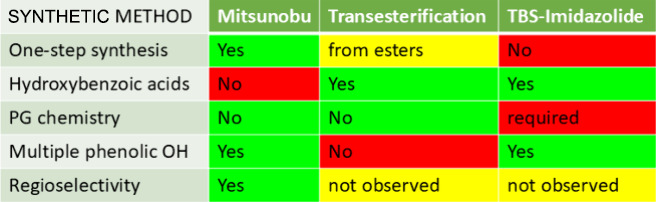

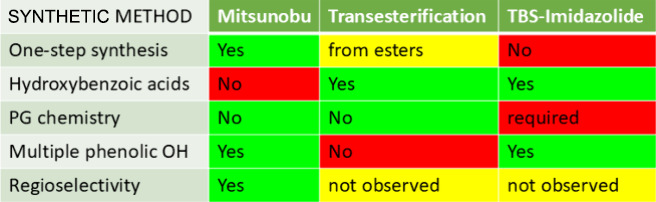

## Introduction

Cellulose is a *β*(1→4)-linked d-glucan and is synthesized by nature in 10^11^–10^12^ t/a scale.^1^ Thus, it is a renewable resource that is excellently suited for sustainable and bioinspired products. Due to the suitability of cellulose in biocompatible systems and its biodegradability, this polymer is appropriate for tailoring of advanced materials in medical applications.^2,3^ Chemical modification of the hydroxyl groups of cellulose yields bioactive derivatives.^4–6^ Due to the covalent linkage of a drug to the polymer backbone, a long-term (non-leaching) activity^7^ and a reduced toxicity^8^ is achieved.

Esterification of cellulose is one of the most versatile derivatization to obtain bio-based materials.^9,10^ For example, cellulose acetate is technically produced in multi-ton quantities.^11^ In addition to industrial procedures, much research about novel reagents, homogenous reactions, and even regioselective modifications was conducted to synthesize tailored derivatives.^10,12^ In particular, phenolic acid esters of cellulose possess antioxidative and antimicrobial properties, which are beneficial for human nutrition, food packaging, and health applications.^13^ They induce increased phosphatase activity, mineralization of bone tissue, and differentiation of osteoblasts. Moreover, membranes composed of e.g. cellulose ferulate are appropriate for applications in hemodialysis.^14^ Despite the fact that phenolic acid esters of polysaccharides occur as structural motifs in plant cell walls, the chemical synthesis of such derivatives is challenging due to phenolic hydroxyl groups.

Most reagents for the activation of carboxylic acids lead to undesired bond formation due to poor discrimination between aliphatic and aromatic hydroxyl groups.^15^ Usually, the required chemoselectivity for the formation of phenolic acid esters is achieved by laborious protecting group chemistry or harsh reaction conditions with an excess of alcohol (e.g., Fischer esterification). The latter method is not suitable for modification of polysaccharides due to polymer degradation by strong acids. Furthermore, reactive double bonds of cinnamic motifs complicate an ester formation due to possible addition reactions with e.g. imidazole.^16^

Approaches to synthesize well-defined products are based on protecting group chemistry, e.g. acetylated phenolic acid chlorides,^17^ or chemoselective reagents such as Steglich and Mitsunobu conditions.^18,19^ On the one hand, Steglich esterification^20^ is still frequently applied, but has several drawbacks such as the sensitizing property of *N*,*N’*-dicyclohexylcarbodiimide (DCC) and the formation of dicyclohexyl urea. This byproduct is hardly removable due to its poor solubility. On the other, Mitsunobu reaction could be used in polysaccharide chemistry for the synthesis of cellulose ethers^21^ and starch benzoates.^22^ Besides its high regio- and stereoselectivity,^23^ Mitsunobu reaction is suitable for chemoselective esterification of phenolic acids and primary alcohols,^15^ which is attractive for cellulose modification.

In our previous article about the Mitsunobu reaction on cellulose, we showed that soluble hydroxycinnamates, i.e. ferulate, coumarate, and caffeate, could be synthesized.^19^ The success of this method is not affected by double bonds or phenolic hydroxyl groups. Thus, it is favorable to synthesize cellulose hydroxycinnamates in one step from the acid. However, Mitsunobu experiments with cellulose and hydroxybenzoic acids are not satisfactory.

Therefore, this work aims to establish a suitable synthesis depending on the character of the phenolic acid. Very recently, the transesterification of cellulose with methyl ferulate was reported,^24^ which is an interesting synthesis path if the methyl ester of the phenolic acid is available. This reaction takes place in a so-called switchable solvent system,^25^ i.e. hydroxyl groups of cellulose are derivatized by CO_2_ in the presence of a base promoting dissolution.^26^

First, it was expected that the transesterification of cellulose with methyl esters in dimethyl sulfoxide (DMSO)/1,8-diazabicyclo[5.4.0]­undec-7-ene (DBU)/CO_2_ can be adapted to various hydroxybenzoates. In practice, the reaction is successful only for monohydroxybenzoates. Cellulose protocatechuate and cellulose gallate could not be synthesized by transesterification. Therefore, in this work, polyphenolic derivatives were synthesized by protecting group chemistry for the first time.

## Experimental Section

### Materials

Microcrystalline cellulose (Avicel PH-101) was purchased from Sigma-Aldrich (product code 11365-1KG) and possessed a ${\overset{®}{\mathrm{DP}}}_{w}$ of 330.^27^ This literature value is based on size exclusion chromatography applying *N*,*N’*-dimethylacetamide (DMA)/LiCl as an eluent. Cellulose possessed a water content of 3.2% and was dried at 60 °C in vacuum before use. Lithium chloride (Carl Roth) was dried at 100°C in vacuum. Cellulose ferulate (DS 0.35) was synthesized by Mitsunobu reaction according to literature.^19^ Other chemicals were purchased from Sigma-Aldrich, VWR, or Carl Roth and were used without further treatment.

### Syntheses

#### Synthesis of Phenolic Acid Esters of Cellulose by Transesterification, General Procedure Adapted from Literature^24^

Cellulose (5 g, 30.8 mmol) was suspended in dry DMSO (70 mL), and 1,8-diazabicyclo[5.4.0]­undec-7-ene (DBU) was added. The mixture was stirred under a CO_2_ atmosphere at 40°C to obtain a clear solution (ca. 0.5 h). Subsequently, the CO_2_ stream was stopped, and the solution was heated to 100°C. During the heat-up phase, the corresponding methyl ester dissolved in DMSO (30 mL) was added. The reaction mixture was stirred for 24 or 48 h under N_2_ flow in the headspace over the solution to remove evolving CO_2_ and methanol. Afterwards, the reaction solution was allowed to cool to room temperature. The product was isolated by precipitation into 2-propanol (2.5 L), filtered off, and washed three times with 2-propanol (0.5 L). The product was dried in vacuum at 40°C to give a yellow solid. For further purification, the triturated material was stirred in DMSO (200 mL) at 40°C over night and poured into 2-propanol, followed by the steps described above.

Example **Cellulose ferulate 2d, peracetylated**

DS = 0.36

FTIR: *ν̃* [cm^–1^] = no *ν̃*(OH), 1741 (C=O)

^1^H NMR (600 MHz, CDCl_3_): *δ* [ppm] = 7.60 (CH), 7.05 (CH), 6.35 (CH), 3.83 (OCH_3_); AGU-CH: 5.00 (1H, 3), 4.73 (1H, 2), 4.34 (2H, 1/6), 3.99 (1H, 6’), 3.64 (1H, 4), 3.47 (1H, 5); 2.25 (CH_3_, ArOAc), 2.06 (CH_3_, 6), 1.94 (CH_3_, 2), 1.87 (CH_3_, 3)

^13^C NMR (150 MHz, CDCl_3_): *δ* [ppm] = 170.3 (6_C=O_, Ac), 169.8 (2_C=O_, Ac), 169.4 (3_C=O_, Ac), 168.8 (C=O, ArOAc), 166.1, 165.5 (C=O), 151.6 (C-OCH_3_), 145.8 (CH), 142.0 (C-OAc), 132.9 (C), 123.5, 121.8, 116.9, 111.5 (CH), 100.6 (C1’), 76.2 (C4), 72.9 (C5), 72.6 (C3), 71.9 (C2), 62.1 (C6), 56.2 (OCH_3_), 20.9 (CH_3_ Ac), 20.8 (CH_3_ ArOAc), 20.7 (CH_3_ Ac), 20.6 (CH_3_ Ac)

#### Protection of Phenolic Acids with *tert*-Butyldimethylsilyl Chloride (TBSCl), General Procedure^28^

The corresponding phenolic acid (30 mmol) was dissolved in dry *N*,*N’*-dimethylformamide (DMF) (75 mL), and imidazole (2.5 equiv per functional group OH, COOH) as well as TMSCl (1.4 equiv per functional group OH, COOH) were added. The reaction mixture was stirred under nitrogen at room temperature overnight. The solution was diluted with 400 mL diethyl ether and washed with water (6×200 mL). The combined extracts were dried over sodium sulfate and evaporated to dryness in vacuum.

The intermediate product was dissolved in THF (450 mL), and diluted acetic acid (600 mL, 3 vol AcOH : 1 vol H_2_O) was added. The reaction mixture was stirred at room temperature overnight. The solution was poured into ice water (1 L), and the product was extracted with ethyl acetate (3×600 mL). If required, then brine was added to achieve phase separation. The combined extracts were washed with brine (3×500 mL), dried over sodium sulfate, concentrated in vacuum, and recrystallized from methanol for purification.

**3,4,5-Tri­[(** ***tert*** **-butyldimethylsilyl)­oxy]­benzoic acid**

Yield: 77 %; Mp. 232-234°C; R_f_ = 0.53 (4:1 cyclohexane/EtOAc)

FTIR (Figure S1): *ν̃* [cm^–1^] = 2931, 2859 (C–H, TBS), 1685 (C=O), 1573 (aromatic)

^1^H NMR (600 MHz, CDCl_3_): *δ* (ppm) = 11.99 (broad), 7.28 (s, 2H), 0.99 (s, 9H), 0.95 (s, 18H), 0.25 (s, 12H), 0.15 (s, 6H)

^13^C NMR (150 MHz, CDCl_3_): *δ* [ppm] = 171.9 (C=O), 148.6 (2xC), 144.2 (C), 121.2 (C), 116.3 (2xCH), 26.33 (2xCH_3_), 26.25 (CH_3_), 19.0 (2xC), 18.7 (C), -3.5 (2xCH_3_), -3.7 (CH_3_)

**3,4-Di­[(** ***tert*** **-butyldimethylsilyl)­oxy]­benzoic acid**

Yield: 80 %; Mp. 150-151°C; R_f_ = 0.39 (4:1 cyclohexane/EtOAc)

FTIR (Figure S2): *ν̃* [cm^–1^] = 2930, 2858 (C–H, TBS), 1682 (C=O), 1598, 1573 (aromatic)

^1^H NMR (600 MHz, CDCl_3_): *δ* (ppm) = 12.02 (broad), 7.62 (dd (8.4 Hz/2.2 Hz), 1H), 7.59 (d (2.2 Hz), 1H), 6.87 (d (8.4 Hz), 1H), 1.00 (s, 9H), 0.99 (s, 9H), 0.24 (s, 6H), 0.23 (s, 6H)

^13^C NMR (150 MHz, CDCl_3_): *δ* [ppm] = 172.0 (C=O), 152.6 (C), 146.9 (C), 124.6 (CH), 122.9 (CH), 122.5 (C), 120.7 (CH), 26.04 (CH_3_), 25.99 (CH_3_), 18.7 (C), 18.6 (C), -3.9 (CH_3_), -4.0 (CH_3_)

**4-[(** ***tert*** **-Butyldimethylsilyl)­oxy]­benzoic acid**

Yield: 63 %; Mp. 109-110°C; R_f_ = 0.63 (2:1 cyclohexane/EtOAc)

FTIR (Figure S3): *ν̃* [cm^–1^] = 2959, 2931, 2858 (C–H, TBS), 1672 (C=O), 1600 (aromatic)

^1^H NMR (600 MHz, CDCl_3_): *δ* (ppm) = 9.71 (broad), 7.88 (pd (8.7 Hz), 2H), 6.75 (pd (8.7 Hz), 2H), 0.85 (s, 9H), 0.10 (s, 6H)

^13^C NMR (150 MHz, CDCl_3_): *δ* [ppm] = 172.1 (C=O), 161.0 (C), 132.5 (CH), 122.4 (C), 120.1 (CH), 25.7 (CH_3_), 18.4 (C), -4.2 (CH_3_)

**4-[(** *tert* **-butyldimethylsilyl)­oxy]-3-methoxybenzoic acid**

Yield: 56 %; Mp. 126-128°C; R_f_ = 0.48 (2:1 cyclohexane/EtOAc)

FTIR (Figure S4): *ν̃* [cm^–1^] = 2927, 2855 (C–H, TBS), 1675 (C=O), 1598, 1580 (aromatic)

^1^H NMR (600 MHz, CDCl_3_): *δ* (ppm) = 11.68 (broad), 7.67 (dd (8.3 Hz/2.0 Hz), 1H), 7.60 (d, (1.9 Hz), 1H), 6.90 (d, (8.2 Hz), 1H), 3.87 (s, 3H), 1.00 (s, 9H), 0.19 (s, 6H)

^13^C NMR (150 MHz, CDCl_3_): *δ* [ppm] = 172.1 (C=O), 150.9 (C), 150.7 (C), 124.4 (CH), 122.7 (C), 120.7 (CH), 113.5 (CH), 55.6 (CH_3_), 25.8 (CH_3_), 18.7 (C), -4.4 (CH_3_)

#### Synthesis of Hydroxybenzoic Acid Esters of Cellulose via TBS-Protected Imidazolides, General Procedure

Cellulose (1 g, 6.17 mmol) was stirred in dry DMA (30 mL) for 2 h at 120 °C. The resulting suspension was allowed to cool to 90 °C, and LiCl (1.8 g) was added to obtain a clear solution.

TBS-protected acid (6.17 or 12.3 mmol) and *N*,*N’*-carbonyldiimidazole (CDI, 6.17 or 12.3 mmol) were dissolved in dry DMA (15 mL) and stirred overnight at 50°C.

This mixture was slowly added to the cellulose solution, stirred overnight at 80°C, and precipitated into deionized water (1 L). The white product was filtered off, washed three times with deionized water (250 mL), and dried under vacuum at 40°C. Purification was achieved by reprecipitation from DMSO, acetone, or 2-butanone.

For deprotection, the material (0.5 g) was dissolved in THF (5 mL) and a tetra-*n*-butylammonium fluoride (TBAF) solution (10 mL, 1 M in THF) was added under stirring. The mixture was heated to 50°C for 2 days and precipitated into ethanol (100 mL). The resulting product was separated, washed three times with ethanol (50 mL), and dried in vacuum at 40 °C. To complete deprotection, the procedure was repeated in DMSO applying TBAF·3H_2_O (2.11 g) and 80°C for 2 d.

Example **Cellulose 4-hydroxybenzoate 3d**

Yield: 56 %; DS = 0.68

FTIR: *ν̃* [cm^–1^] = 3370 (OH), 2888 (CH), 1700 (C=O), 1608, 1593 (C=C)

^1^H NMR (600 MHz, DMSO-d6): *δ* (ppm) = 10.29 (1H, OH), 7.87 (2H, CH), 6.83 (2H, CH), 6.0-2.7 (AGU)

^13^C NMR (150 MHz, DMSO-d6): *δ* (ppm) = 165.6 (C=O), 162.5 (C), 132.2 (CH), 120.6 (C), 115.8 (CH), 103.1 (C1), 80.3, 75.2, 73.5 (C2-5)

#### Determination of DS Values from ^1^H NMR Spectra of Peracetylated Products

The phenolic acid ester of cellulose (300 mg) was suspended in pyridine (5 mL), and acetic anhydride (5 mL) was added. The reaction mixture was stirred at 60 °C overnight. Afterward, undissolved impurities were removed by centrifugation. The product was isolated by precipitation into deionized water (150 mL) containing NaHCO_3_ (0.5 g) and subsequent filtration. For further purification, the material was washed three times with deionized water (100 mL) and dried in a vacuum at 40 °C.

DS values were calculated from integral intensities (I) of ^1^H NMR signals of aromatic CH-protons (8.5–6.0 ppm) and CH_3_-protons (acetate, 2.5–1.5 ppm) according to the following equations:

1 $$DS_{ferulate}=\frac{9I_{CH}}{5I_{CH_{3}}}$$

2 $$DS_{4-hydroxybenzoate}=\frac{9I_{CH}}{4I_{CH_{3}}}$$

3 $$DS_{vanillate}=\frac{3I_{CH}}{I_{CH_{3}}}$$

4 $$DS_{protocatechuate}=\frac{3I_{CH}}{I_{CH_{3}}-I_{CH}}$$

#### Determination of DS Values by Gravimetry

Crucibles were pretreated with concentrated hydrochloric acid, rinsed with deionized water, and heated to 800°C for 4 h. After applying gravimetric standard procedures, cellulose derivatives (100 mg) were allowed to react with oleum (2 mL, 20 % SO_3_) in covered crucibles at room temperature overnight. Afterward, the crucibles were gently heated (no boiling) with a gas burner until the samples were solidified. The crucibles were further heated until evolution of fumes stopped before they were placed into the muffle furnace. The calculation of the DS values was based on the percentage by mass of SiO_2_ according to the following equation:

5 $$SiO_{2}\left[\%\right]=\frac{DS\times f\times M_{SiO_{2}}\times 100}{M_{AGU}+DS\times M_{S}}$$

M_S_ is defined as the net molar mass increase by the substituent, and f is the number of TBS groups of the phenolic acid. M_AGU_ is the molar mass of an anhydroglucose unit.

### Measurements

#### NMR Spectroscopy

NMR spectra were acquired on a Bruker Avance III 600 MHz spectrometer applying up to 100 mg mL^–1^ sample in CDCl_3_ or DMSO-d6. Relaxation delay was set to 10 s for the quantitative evaluation of the ^1^NMR measurements.

#### FTIR Spectroscopy

FTIR spectra were measured on a Tensor27 instrument (Bruker Optics GmbH) by the ATR technique.

#### Size Exclusion Chromatography

SEC measurements were performed with an Azura HPLC/UHPLC unit (Knauer) with a UV detector at 254 nm applying DMSO with 0.1% NaNO_3_ and a sample concentration of 3 mg mL^–1^. The set temperature was 60 °C and the flow rate was 0.3 mL min^–1^ at 44 bar. PolarGel-M (Agilent) and ABOA DMSO-phiL-P-250 (AppliChrom) columns were applied for separation. Dextran standards in the range of 180–225000 g mol^–1^ were used for calibration. The samples were filtered through a syringe filter (0.45 *μ*m) with a PTFE membrane before injection (30 *μ*L).

## Results and Discussion

### Synthesis of Phenolic Acid Esters of Cellulose by Transesterification

In a first set of experiments, the state of the art was reproduced by applying slight variations. According to Figure 1, cellulose was dissolved in DMSO/DBU/CO_2_ and allowed to react with methyl ferulate at 100°C for 24 or 48 h. As indicated by Libretti et al., a high amount of DBU was used, which is beneficial for the transesterification if phenolic groups are present.^24^ Due to the acidity of phenolic hydroxyl groups, enough DBU is required to deprotonate such OH moieties and perform the reaction. The DS values of obtained cellulose ferulates increased with increasing molar ratio of reagent to anhydroglucose unit and reaction time (Table 1). Product **2b** (DS 0.20) was synthesized with 3 eq. methyl ferulate and 6 eq. DBU per repeating unit by applying a reaction time of 48 h. In the previous study,^24^ the DS value observed under these conditions was 0.29. However, this discrepancy can be explained by the lower cellulose concentration of 2.3 wt % in the present work compared to 3.3 wt % indicated in the literature.^24^ It was encouraging that using 6 eq. methyl ferulate and 9 eq. DBU per repeating unit led to product **2d** with the highest DS value of 0.36, which could not be achieved in previous studies up to now.

**Figure 1:**
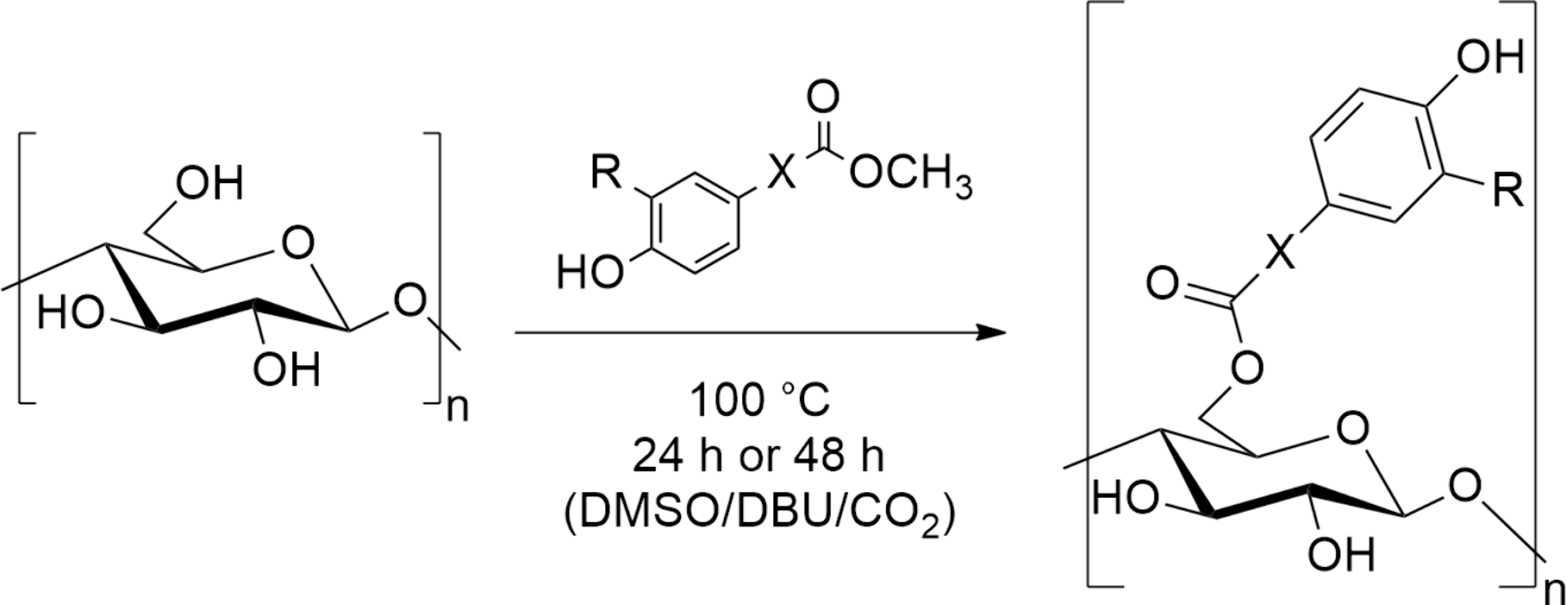
Reaction scheme for the synthesis of phenolic acid esters of cellulose by transesterification. Ferulate: X = CH=CH, R = OCH_3_; 4-Hydroxybenzoate: X = −, R = H; Vanillate: X = −, R = OCH_3_.

**Table 1:** Synthesis of the Phenolic Acid Esters of Cellulose by Transesterification

Phenolic acid^a^	eq ester^b^	eq DBU^c^	Time [h]	No	DS^d^	AE [%]^e^	E-factor^f^
Ferulic acid	3	6	24	**2a**	0.16	97	3.1
Ferulic acid	3	6	48	**2b**	0.20	97	3.0
Ferulic acid	6	9	24	**2c**	0.22	97	6.0
Ferulic acid	6	9	48	**2d**	0.36	95	5.3
4-Hydroxybenzoic acid	3	6	48	**3a**	0.14	98	2.5
4-Hydroxybenzoic acid	6	9	24	**3b**	0.12	98	5.2
4-Hydroxybenzoic acid	6	9	48	**3c**	0.14	98	5.0
Vanillic acid	3	6	24	**4a**	0.08	99	3.1
Vanillic acid	6	9	24	**4b**	0.09	98	6.1
Vanillic acid	6	9	48	**4c**	0.11	98	6.0
Protocatechuic acid	3	9	24	**5a**	-^g^	-	-
Protocatechuic acid	6	15	24	**5b**	-^g^	-	-
Gallic acid	3	12	24	**6a**	-^g^	-	-
Gallic acid	6	21	24	**6b**	-^g^	-	-

a Methyl ester applied for synthesis

b Mole ester per mole anhydroglucose unit.

c Mole 1,8-diazabicyclo[5.4.0]­undec-7-ene per mole anhydroglucose unit.

d Degree of substitution, determined by ^1^H NMR spectroscopy after peracetylation.

e Atom economy (consider SI).

f Environmental factor (consider SI).

g No conversion.

In further experiments, methyl esters of hydroxybenzoic acids were applied. 4-Hydroxybenzoate yields products **3a**–**c**, possessing DS values of 0.12 to 0.14. Cellulose vanillates **4a**–**c** could be obtained with comparatively low DS values of 0.08 to 0.11. In general, the DS of hydroxybenzoates **3-4** depends on molar ratio and reaction time as observed for cellulose ferulates, and is lower than the DS values of **2a**–**d**. No conversion was observed for protocatechuic acid ester and methyl gallate. Phenolic acid esters obtained by transesterification were completely soluble in DMSO if the DS value was ≥0.2.

The signals of FTIR- and NMR spectra of cellulose ferulates obtained by transesterification are in accordance with the literature.^19,24^ Peracetylation of the ferulates leads to a higher resolution of the spectra and reliable determination of the DS values from integral intensities of ^1^H NMR experiments (Figure 2, bottom). Resonances of the ^13^C NMR spectrum (Figure 2, top) could be completely assigned by means of the HSQC DEPT (Figure S5). The carbonyl signal (7) at 166 ppm shows successful ester formation on the cellulose backbone. The chemical shifts of cellulose acetate-like structures are well known from the literature.^29^ The molecular structure, including the DS values, of cellulose hydroxybenzoates and -vanillates was revealed similarly (Figures S6 and S7).

**Figure 2:**
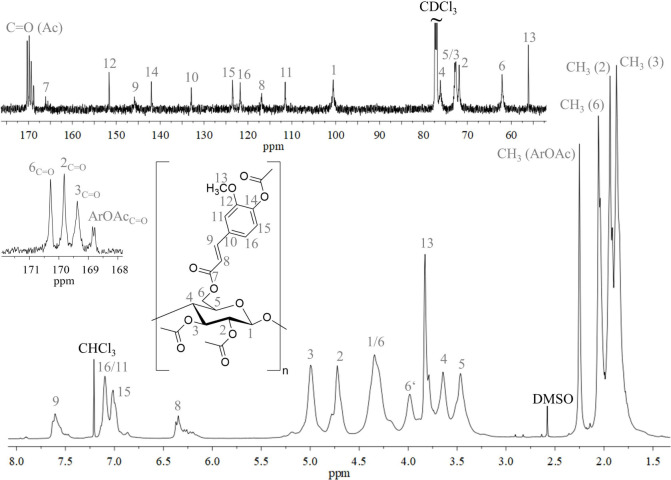
Sections of NMR spectra (top: ^13^C, bottom: ^1^H) of peracetylated cellulose ferulate **2d** recorded in CDCl_3_.

In contrast to well exploited conversions of polysaccharides with vinyl esters under mild conditions,^10^ transesterifications of more practical methyl esters require high temperatures around 100°C. In early attempts, concentrations and temperatures were already optimized but based on potassium methoxide as catalyst.^30^ In recent studies, 1,5,7-triazabicyclo[4.4.0]­dec-5-ene (TBD) was used either in 1-butyl-3-methylimidazolium chloride (BMIMCl)^31^ or in the switchable solvent system DMSO/TBD/CO_2_.^32^ In this context, the present transesterification of cellulose with aromatic methyl esters in DMSO/DBU/CO_2_ is favored, since recycling of 2-propanol, DMSO, and DBU could be demonstrated in a previous study.^24^

Moreover, syntheses were evaluated regarding the atom economy (AE) and environmental factor (E-factor) (Table 1). Therefore, approximations of Onwukamike et al.,^33^ e.g. solvent recovery and 100% yield, were applied to discuss best-case green chemistry metrics recommended for homogeneous modification of cellulose (consider SI). In general, AE is very high (95 to 99 %) for the transesterification, due to the only byproduct methanol and low DS values. However, E-factors are in the upper range (2.5-6.1) especially for high molar ratios, which did not result in higher DS values.

In light of harsh reaction conditions in transesterification reactions (100°C for 48 h), which may lead to depolymerization of cellulose, the molecular weight distribution was determined by SEC. In fact, the molecular weight of cellulose ferulate obtained by the Mitsunobu reaction was lower than the mass of ferulate **2d**. The Mitsunobu product (DS_FA_ = 0.35) possessed a *M*_w_ of 80230 g mol^–1^ (PDI 3.0), but derivative **2d** (DS_FA_ = 0.36) possessed a *M*_w_ of 70272 g mol^–1^ (PDI 3.1), starting from the same microcrystalline cellulose. However, the degradation of the polymer chains was not strongly pronounced.

In comparison to the starting material cellulose, the apparent DP_w_ of derivative **2d** decreased from 330 (untreated cellulose) to 311 but increased from 330 to 358 for the Mitsunobu product. This result could be explained by deviations in the polymer structures from the SEC standard or different solution states. Moreover, the molecular weight distribution could be increased by cross-linking of ferulate moieties as observed in plant cell walls of grasses.^34^

Libretti et al. performed transesterification with methyl ferulate in absence of cellulose to exclude the formation of phenolic oligomers by ^1^H NMR spectroscopy.^24^ In this work, the formation of oligomers during the reaction was excluded by thin-layer chromatography of an extract obtained from the blank test.

### Synthesis of Phenolic Acid Esters of Cellulose via *tert*-Butyldimethylsilyl (TBS)-Protected Imidzolides

To synthesize cellulose protocatechuate and gallate, as well as hydroxybenzoates with higher DS values, protecting groups were necessary. The phenolic hydroxyl groups of 4-hydroxybenzoic acid, vanillic acid, protocatechuic acid, and gallic acid were modified with TBS moieties applying a known two-step procedure.^28^ Afterwards, protected acids were converted with *N*,*N’*-carbonyldiimidazole (CDI) to yield the corresponding imidazolides (Figure 3). The activated carboxylic acids were allowed to react with cellulose dissolved in DMA/LiCl at 80°C for 24 h. This esterification procedure is known to be very efficient in absence of phenolic hydroxyl groups and reactive double bonds.^35^ The obtained products were soluble in easily evaporable solvents, such as tetrahydrofuran (THF), 1,4-dioxane, acetone, and 2-butanone. However, they were insoluble in chloroform and ethyl acetate.

**Figure 3:**
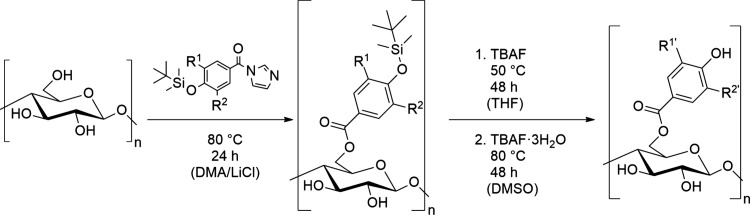
Reaction scheme for the synthesis of hydroxybenzoic acid esters of cellulose via TBS-protected imidazolides. 4-Hydroxybenzoate: R^1^ = R^2^ = H; Vanillate: R^1^ = OCH_3_, R^2^ = H; Protocatechuate: R^1^ = OTBS, R^2^ = H; Gallate: R^1^ = R^2^ = OTBS; R^1’^ and R^2’^ indicate deprotected form (OH) where applicable.

The desilylation of the products with tetra-*n*-butylammonium fluoride (TBAF) had to be carried out twice to complete the deprotection. In practice, the procedure was adapted from the desilylation of *tert*-butyldimethylsilyl methylcellulose.^36^ Thus, the first deprotection step could be performed in THF/TBAF. After this treatment, the derivatives were not soluble in THF anymore but in DMSO. This solvent was used for complete deprotection in DMSO/TBAF. Attempts to deprotect the derivatives just in one step in DMSO/TBAF were not convenient and lead to more dark colored by-products.

In addition to the removal of TBS groups, cleavage of the ester bond can be expected. The deacylation of cellulose acetate takes place in THF/TBAF or DMSO/TBAF^37^ according to the ketene (E1cB) mechanism at positions 2 and 3.^38^ The ester cleavage at position 6 is slow and follows the general base-catalyzed mechanism. However, cellulose esters without *α*-protons (e.g. benzoate) were deacylated, too.^39^ It could be assumed that phenolic cellulose esters (no *α*-proton) were partially deacylated under the conditions in the present study.

Finally, products with DS values between 0.19 and 0.94 could be obtained at low molar ratios of 1 or 2 (Table 2). Moreover, this method permitted the synthesis of cellulose benzoates possessing multiple hydroxyl groups. Cellulose protocatechuate (**5c**, DS 0.38) and cellulose gallate (**6c**, DS 0.94) could be obtained, which is not possible by other synthetic methods up to now.

**Table 2:** Synthesis of Hydroxybenzoic Acid Esters of Cellulose via TBS-Protected Imidazolides

Phenolic acid^a^	eq imidazolide^b^	No	DS	AE [%]^c^	E-factor^d^
4-Hydroxybenzoic acid	2	**3d**	0.68^e^	55	5.2
Vanillic acid	1	**4d**	0.19^e^	77	3.6
Vanillic acid	2	**4e**	0.53^e^	61	5.5
Protocatechuic acid	2	**5c**	0.38^e^	58	9.7
Gallic acid	2	**6c**	0.94^f^	38	9.1

a Phenolic OH groups substituted with TBS.

b Mole imidazolide (TBS-protected) per mole anhydroglucose unit.

c Atom economy (consider SI).

d Environmental factor (consider SI).

e Degree of substitution, determined by ^1^H NMR spectroscopy after peracetylation of deprotected product.

f Degree of substitution, determined by gravimetry of TBS-protected product.

Figure 4 shows typical ^1^H NMR spectra of cellulose hydroxybenzoates obtained via TBS-protected imidazolides. The esterification reaction and one-step deprotection of the products lead to partially TBS-protected derivatives. In the ^1^H NMR spectrum (Figure 4, top), the phenolic hydroxyl groups (Ar–OH) at around 10–11 ppm are already visible. However, TBS groups are indicated in a high field. These partially protected phenolic structures lead to overlapping CH resonances of the aromatic ring between 6.0 and 8.5 ppm. Just after the second deprotection step in DMSO/TBAF at 80°C, a fully deprotected cellulose benzoate was obtained. No signals arising from TBS groups are visible (Figure 4, bottom). The linkage to the polymer backbone is clearly indicated by the C=O signal at 165 ppm in the ^13^C NMR spectrum (Figure S9).

**Figure 4:**
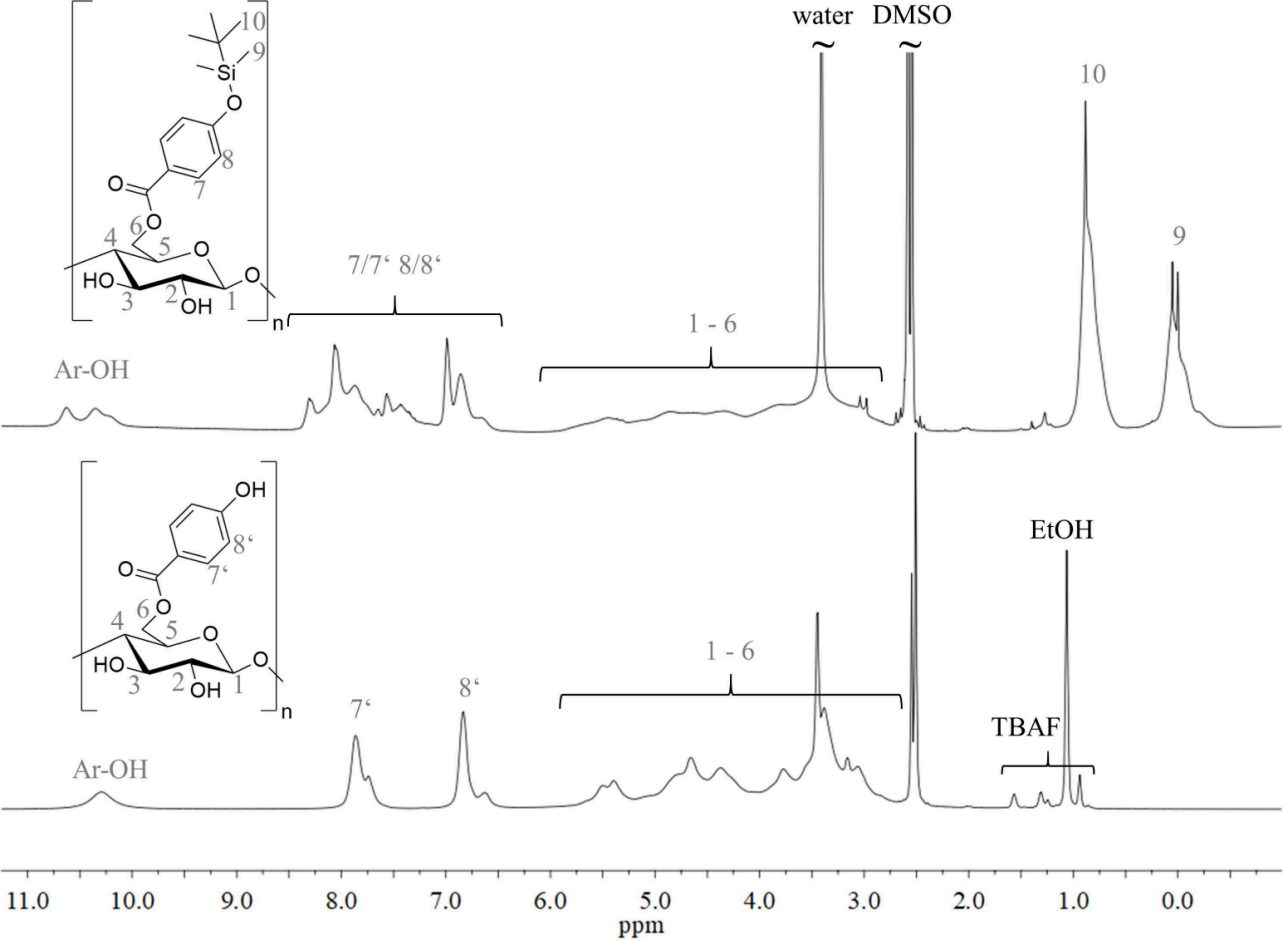
^1^H NMR spectra of cellulose hydroxybenzoate **3d** (top: partially TBS-protected; bottom: fully deprotected) recorded in DMSO-d6.

The success of the deprotection steps could also be confirmed by FTIR spectroscopy. The C–H vibrations below 3000 cm^–1^ indicate whether the deprotection was completed. The sharp peaks arising from TBS moieties are not present, and only one broad signal from the C–H stretch of cellulose remains (Figure S10).

The DS values were determined by ^1^H NMR spectroscopy of the peracetylated products as described above. However, cellulose gallate (**6c**) could not be acetylated, which might be explained by the formation of stable intramolecular hydrogen bonds of multiple phenolic hydroxyl groups.^40,41^ Thus, the DS value of the TBS-protected compound was determined gravimetrically as SiO_2_ after decomposition with oleum.^42^ It must be noted that the amount of TBS groups was quantified by ^1^H NMR spectroscopy since some moieties are removed already in the first synthesis step (esterification). In average, there were two TBS groups per gallate moiety, which was considered in the calculation (eq 5). However, DS determination prior to deprotection steps is more susceptible to errors since ester cleavage is possible during the treatment with TBAF. Thus, a lower DS value of **6c** could be assumed.

For synthesis with TBS-protected imidazolides, AE is inherently lower compared to transesterification, e.g., 38% for cellulose gallate **6c** (Table 2). AE is increased for phenolic acids possessing only one hydroxyl/TBS group and low DS values, e.g., 77% AE for cellulose vanillate **4d**. Significant differences could be also observed for E-factors. The syntheses of cellulose protocatechuate and cellulose gallate possess high values between 9 and 10. For monohydroxybenzoates, E-factors are in the range of 3.6 to 5.5, like values of transesterification. However, only the reactions involving cellulose were considered. The synthesis of TBS-protected phenolic acids leads probably to higher E-factors than synthesis of methyl esters.

## Conclusions

In our previous study, the Mitsunobu reaction was applied for the synthesis of cellulose hydroxycinnamates and found to be superior compared with other methods. The advantages of a one-step synthesis (no protecting groups necessary) and conversion of polyphenolic moieties were highlighted. However, the procedure was not suitable to yield cellulose hydroxybenzoates. In this work, phenolic cellulose esters were synthesized either by transesterification of the biopolymer with methyl esters or by conversion with TBS-protected imidazolides. At first, the transesterification of cellulose with methyl ferulate in DMSO/DBU/CO_2_ was adapted to synthesize cellulose 4-hydroxybenzoate and cellulose vanillate. This one-step procedure starting from the methyl ester turned out to be suitable for phenols possessing only one hydroxyl group. The DS values of the obtained cellulose hydroxybenzoates were relatively low (DS 0.08 to 0.14). Conversion of protocatechuate and gallate methyl esters was not successful. Therefore, esterification of TBS-protected phenolic acids prepared by means of *N*,*N’*-carbonyldiimidazole was applied, which yields the cellulose esters of polyphenols and products with higher DS values of up to 0.94 for cellulose gallate.

To summarize, synthetic methods were developed to synthesize phenolic acid esters of cellulose that have pros and cons. However, three recommendations can be given: 1) For the synthesis of cellulose hydroxycinnamates, Mitsunobu chemistry is preferred. 2) Transesterification in DMSO/DBU/CO_2_ may yield hydroxybenzoates possessing only one phenolic hydroxyl group. 3) TBS-protected imidazolides lead to products with multiple phenolic hydroxyl groups.

Phenolic acid esters of cellulose show antioxidative and antimicrobial properties with long-term activity and reduced toxicity (non-leaching). Therefore, they are promising for applications in medicine, e.g., wound dressings and regenerative therapies. Due to their solubility in organic solvents, the products could be shaped into advanced materials such as nanometric films, particles, and fibers, which will be a subject of further studies.

## Supplementary Material



## Notes

FTIR and NMR spectra for sample characterization (PDF)
