# Supplementary material for: Exploring the Syntheses for Phenolic Acid Esters of Cellulose: Pros and Cons of Methods
Source: Polym Sci Technol. 2025 Apr 29;1(9):779–86. doi: 10.1021/polymscitech.5c00028 (PMC13052622; doi:10.1021/polymscitech.5c00028)
Supplement: Supplementary file 1 [file ps5c00028_si_001.pdf]

Supporting information

Exploring the syntheses for phenolic acid esters of cellulose - pros and cons of methods

Thomas Elschner*, Jakob Schönrich, and Steffen Fischer

Institute of Plant and Wood Chemistry, Technische Universität Dresden, Pienner Str. 19, 01737 Tharandt, Germany

Email: thomas.elschner@tu-dresden.de

Figure S1:

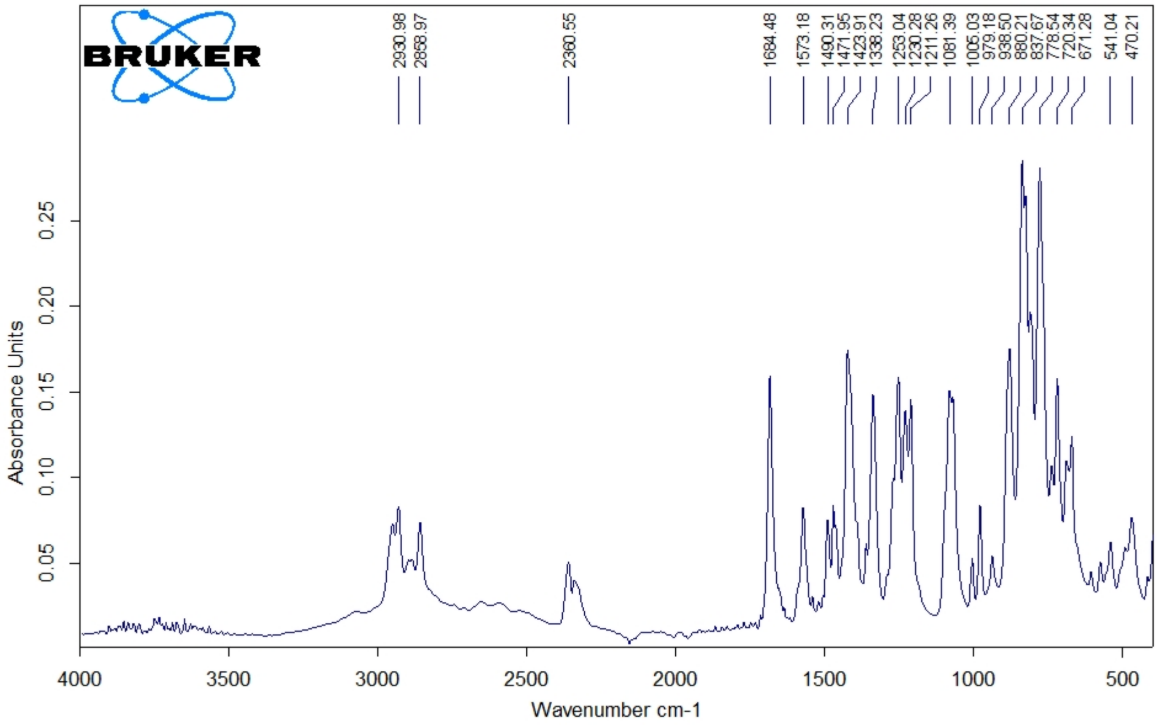

FTIR spectrum of 3,4,5-tri[(tert-butyldimethylsilyl)oxy]benzoic acid.

Figure S2:

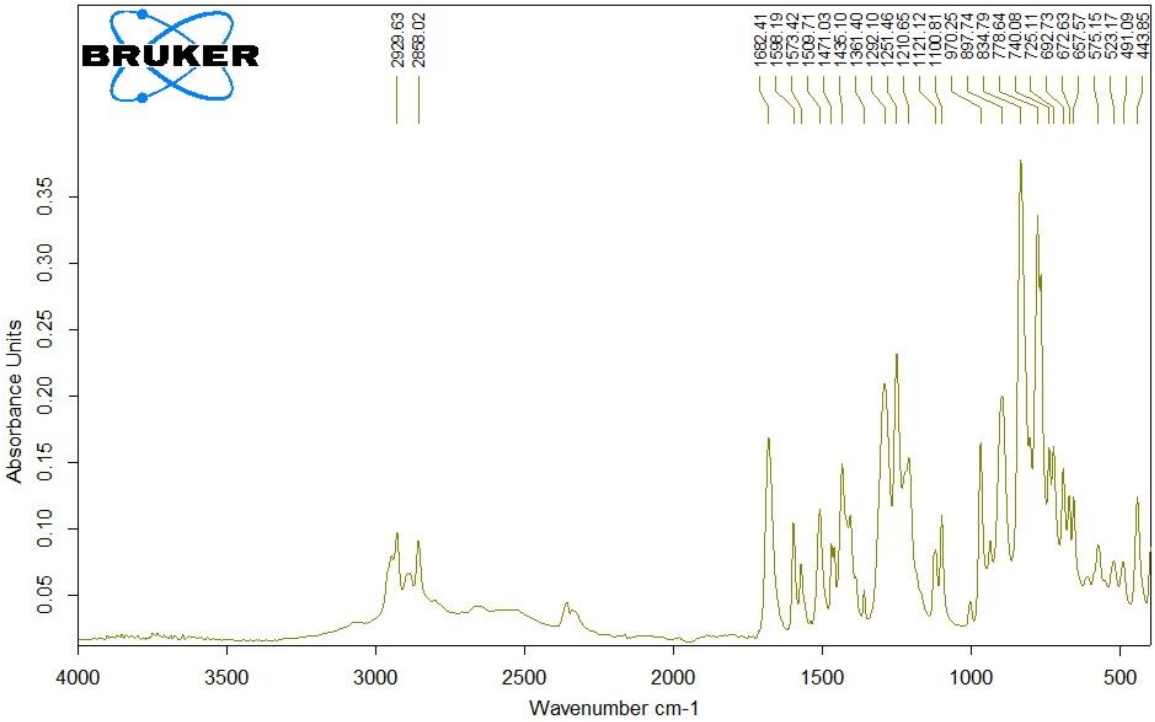

FTIR spectrum of 3,4-di[(tert-butyldimethylsilyl)oxy]benzoic acid.

Figure S3:

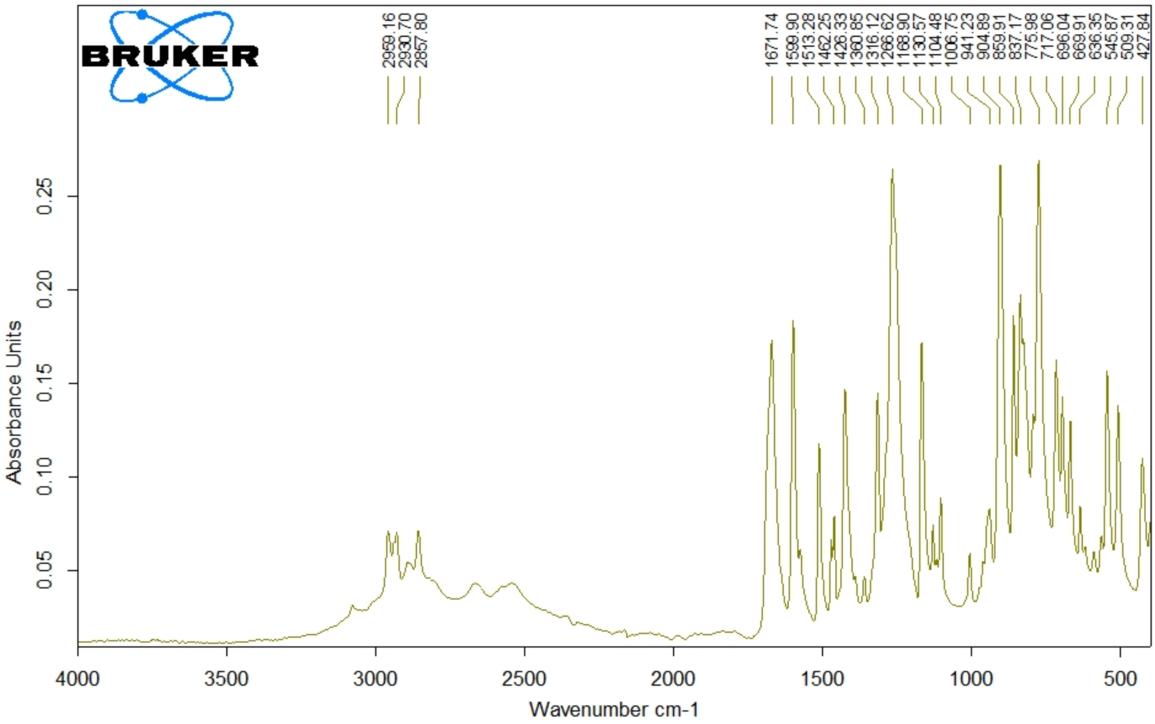

FTIR spectrum of 4-[(tert-butyldimethylsilyl)oxy]benzoic acid.

Figure S4:

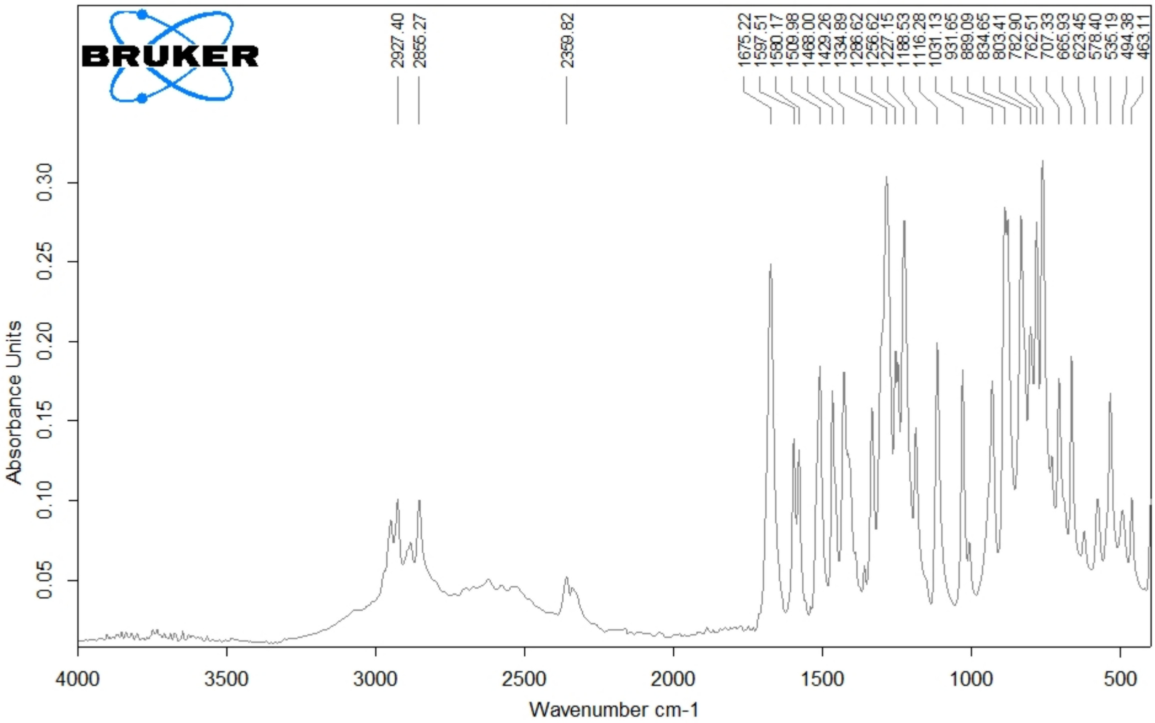

FTIR spectrum of 4-[(tert-butyldimethylsilyl)oxy]-3-methoxybenzoic acid.

Figure S5:

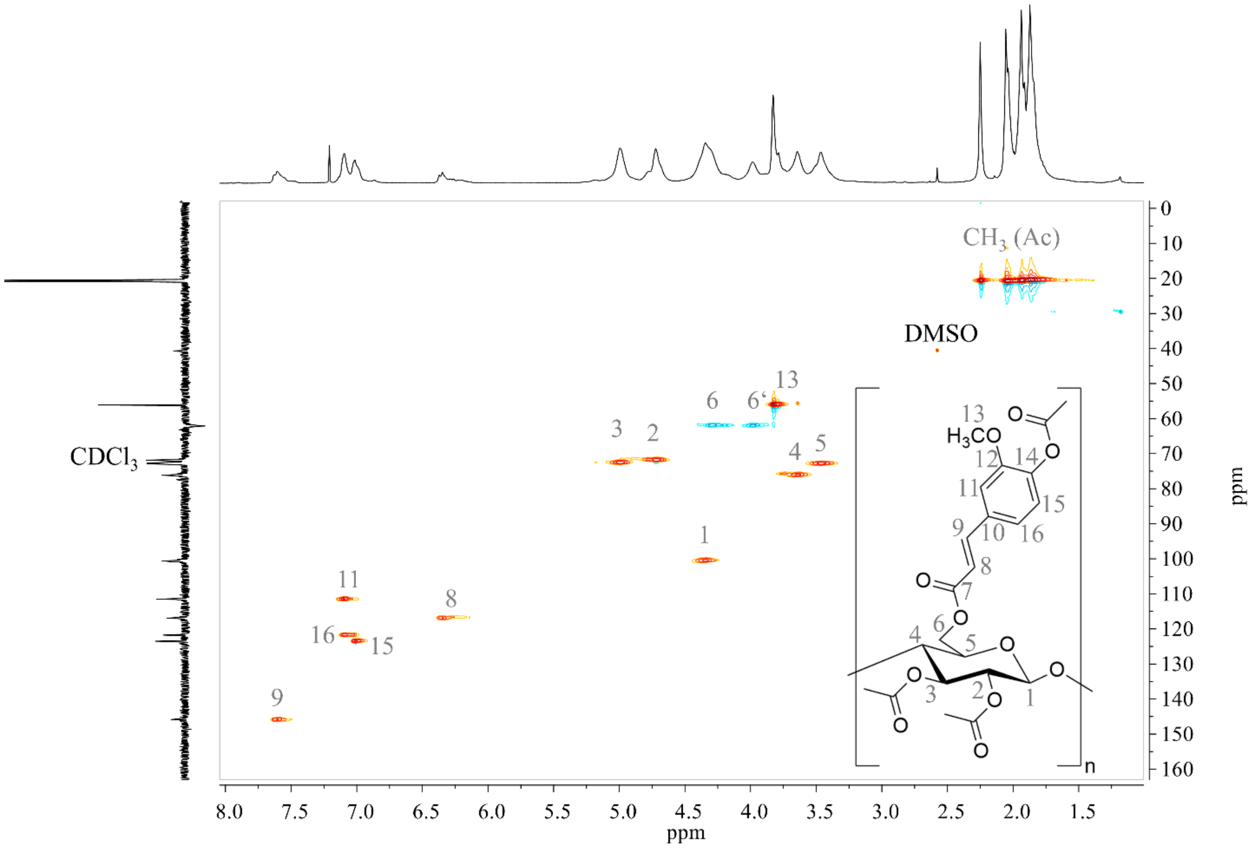

HSQC DEPT NMR spectrum of peracetylated cellulose ferulate **2d** recorded in $CDCl_3$.

Figure S6:

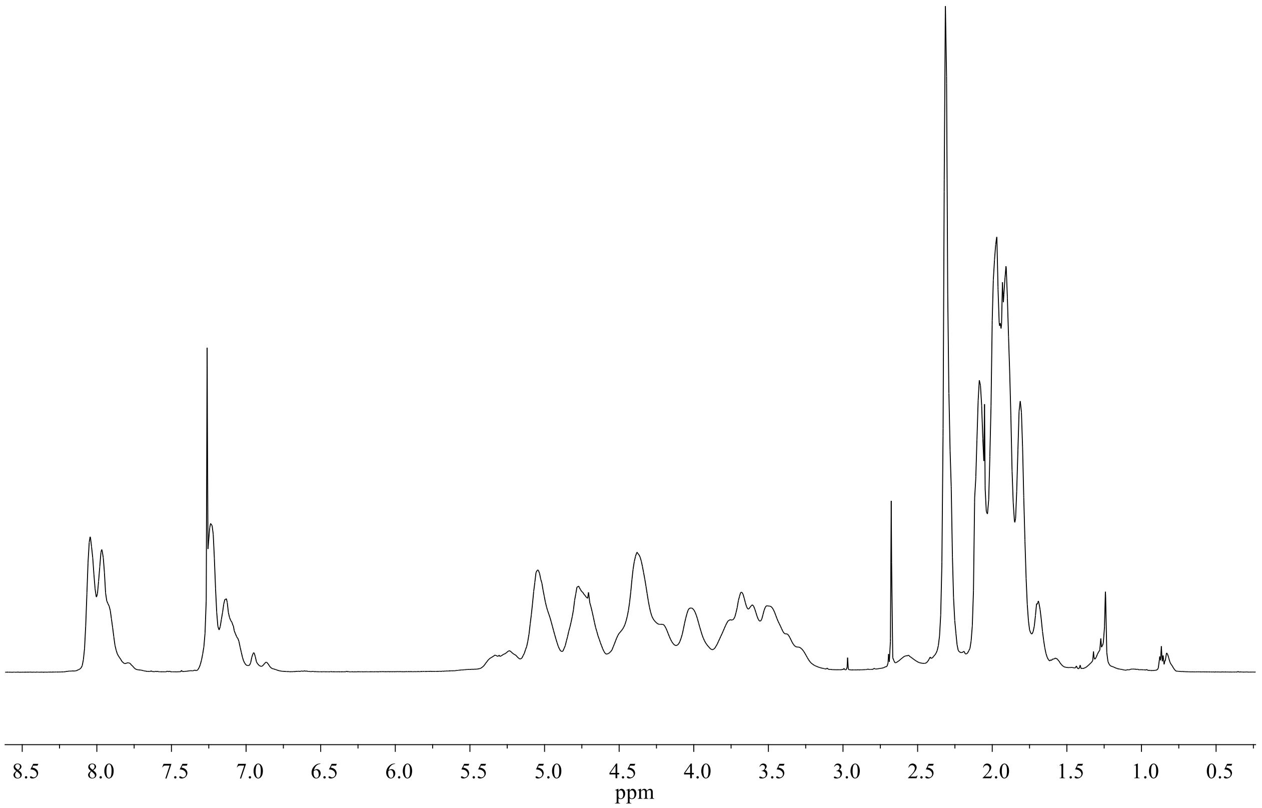

^{1}H NMR spectrum of peracetylated cellulose hydroxybenzoate **3d** recorded in $CDCl_3$.

Figure S7:

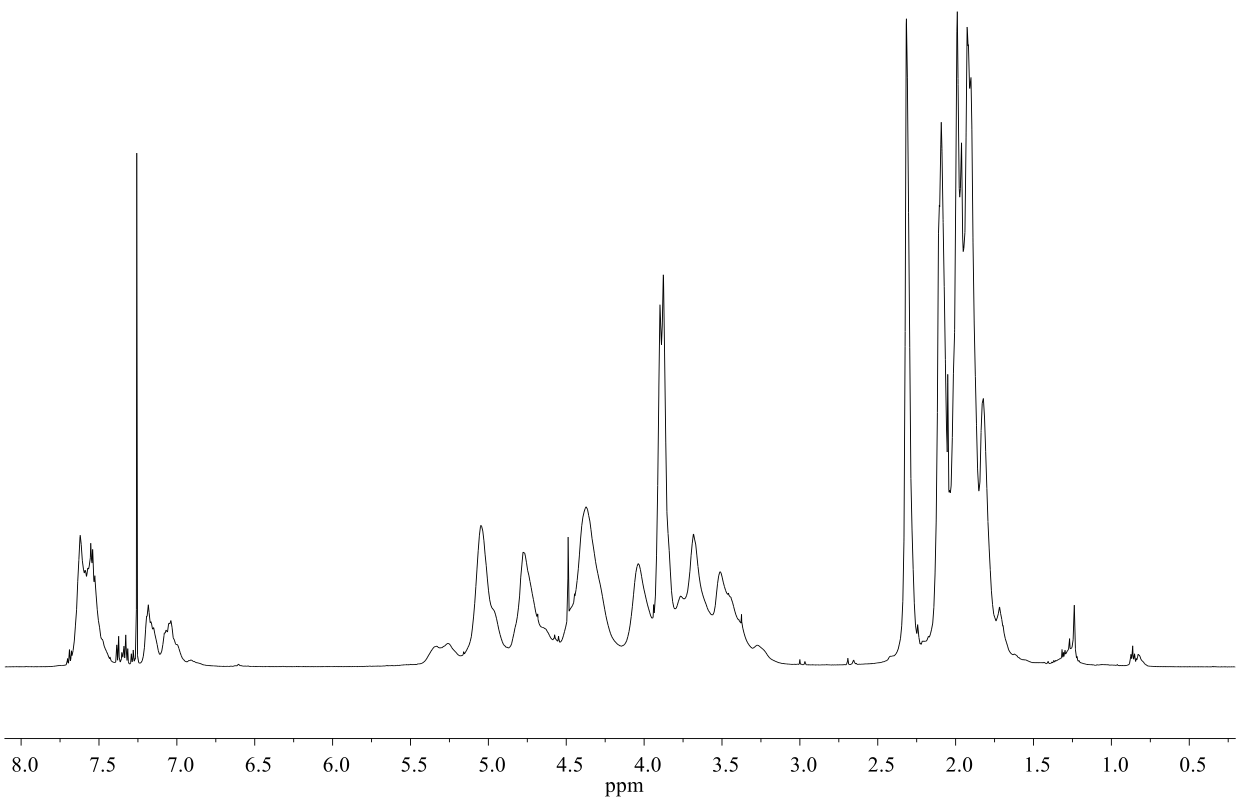

^{1}H NMR spectrum of peracetylated cellulose vanillate **4e** recorded in $CDCl_3$.

Figure S8:

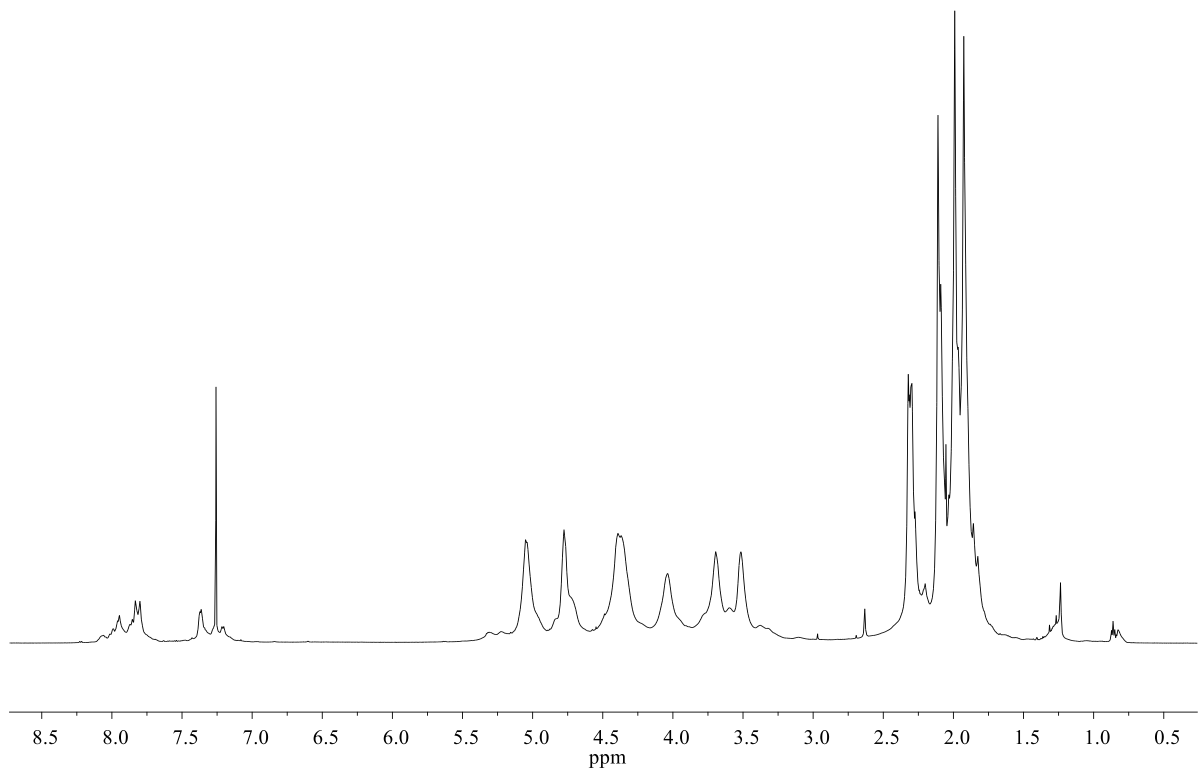

^{1}H NMR spectrum of peracetylated cellulose protocatechuate **5c** recorded in $CDCl_3$.

Figure S9:

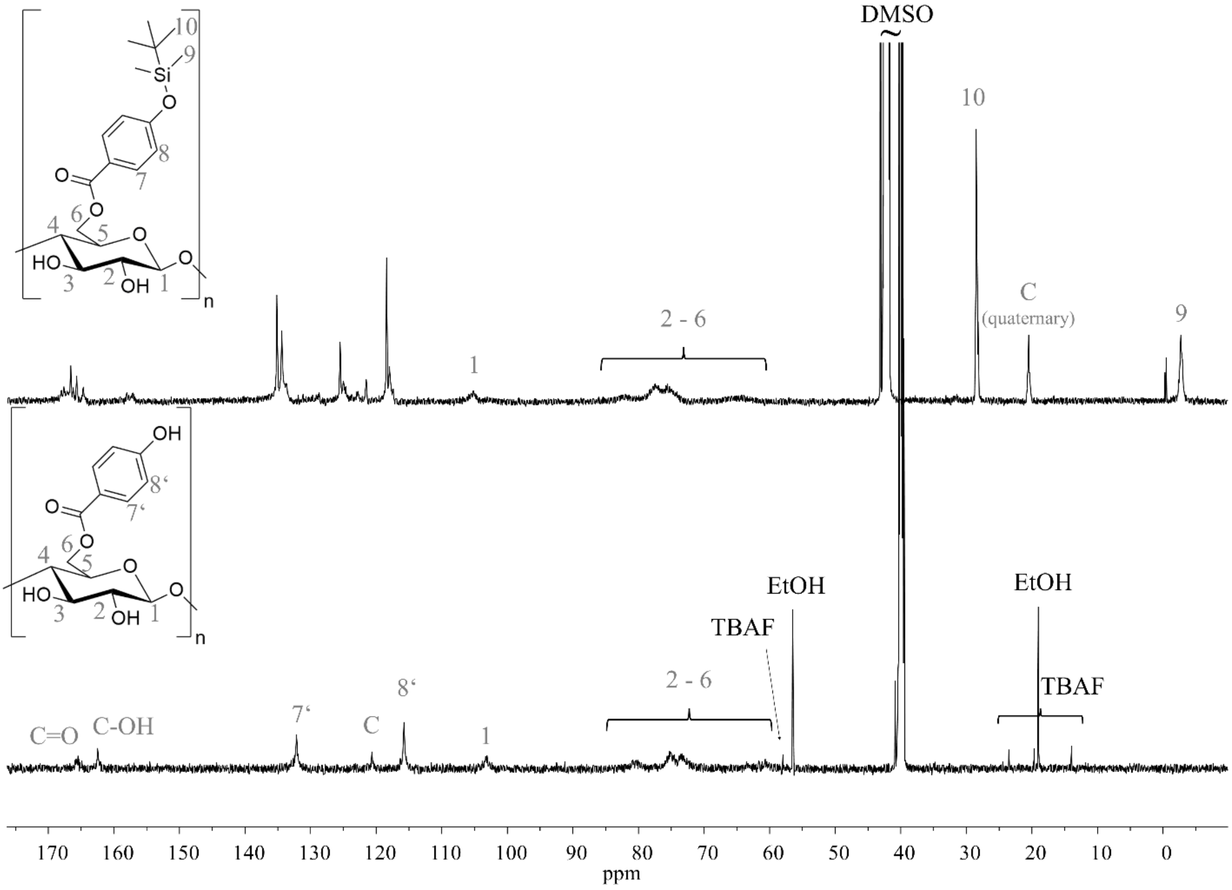

^{13}C NMR spectra of cellulose hydroxybenzoate **3d** (top: partially TBS-protected, bottom: fully deprotected) recorded in DMSO-d6.

Figure S10:

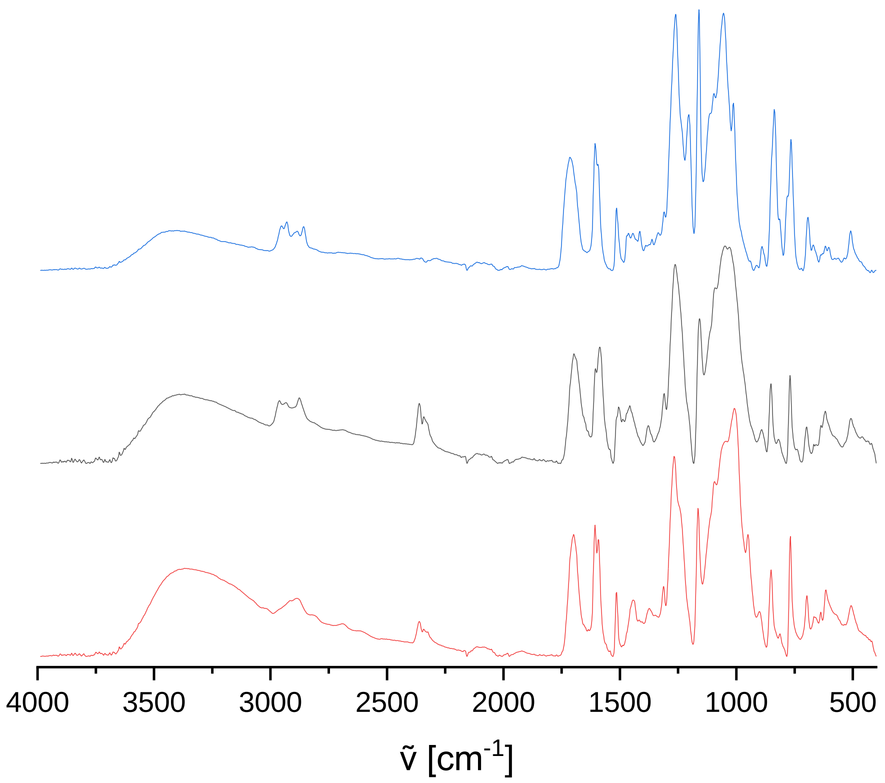

FTIR spectra of cellulose hydroxybenzoate **3d** (top: TBS-protected, center: after first deprotection step, bottom: fully deprotected after the second deprotection step).

Calculation of atom economy (AE):

Eq. S1: AE(transesterification) [%] = $\frac{M\ (repeating\ unit\ of\ product)*100}{M(anhydroglucose\ unit)+DS*M(methyl\ ester)}$

Eq. S2: AE(TBS-imidazolide) [%] = $\frac{M\ (repeating\ unit\ of\ product)*100}{M(anhydroglucose\ unit)+DS*M(CDI)+DS*M(TBSacid)}$

Calculation of environmental factor (E-factor):

The lowest possible E-factor was determined by the following equations assuming recovery of solvents (100 %) and TBAF as well as 100 % yield. Synthesis of starting materials and purification steps were not considered. Calculations are based on synthesis with 1 g (6.17 mmol) of cellulose.

Eq. S3:

E-factor (transesterification) = $\frac{[(1+M(methyl\ ester)*Eq.ester*0.0617)]-[(M(repeating\ unit\ of\ product)*0.0617)]}{[(M(repeating\ unit\ of\ product)*0.0617)]}$

Eq. S4:

E-factor (TBS-imidazolide) =
$\frac{[(1+M(CDI)*Eq.*0.0617+M(TBSacid)*Eq.*0.0617+M(TBAF)*Eq.*0.0617*number\ of\ TBS)]-[(M(unit)*0.0617)]}{[(M(unit)*0.0617)]}$
